# The Use of the Bioelectrical Impedance Phase Angle to Assess the Risk of Sarcopenia in People Aged 50 and above in Poland

**DOI:** 10.3390/ijerph19084687

**Published:** 2022-04-13

**Authors:** Małgorzata Kołodziej, Sławomir Kozieł, Zofia Ignasiak

**Affiliations:** 1 Department of Biostructure, Wroclaw University of Health and Sport Sciences, 51-612 Wrocław, Poland; zofia.ignasiak@awf.wroc.pl; 2 Department of Anthropology, Hirszfeld Institute of Immunology and Experimental Therapy, Polish Academy of Sciences, 50-449 Wrocław, Poland; slawomir.koziel@hirszfeld.pl

**Keywords:** phase angle, pre-sarcopenia, appendicular skeletal muscle, muscle quality, healthy aging

## Abstract

Purpose: The increasing aging of many populations requires a continuous evolution of assessment methods in geriatrics, especially methods for identifying sarcopenia. Early diagnosis of unfavorable changes in the condition of skeletal muscles and the implementation of therapeutic methods may reduce the risk of functional limitations in the elderly. The aim of the study was to evaluate the association between the bioelectrical impedance phase angle and the occurrence of pre-sarcopenia in people aged 50 and above. Methods: 1567 people aged 50–87 were examined. Anthropometric as well as muscle strength and walking speed measurements were performed. Using bioelectrical impedance analysis, the phase angle was measured and the appendicular skeletal muscle mass was estimated. The contribution of the phase angle in explaining the probability of the occurrence of pre-sarcopenia was verified by multivariate logistic regression. Results: Sarcopenia was diagnosed in 12 people (0.8%) and pre-sarcopenia in 276 people (17.6%). Significantly lower impedance phase angle and muscle functional quality were found in people with confirmed pre-sarcopenia compared to people without sarcopenia. The relative differences for the phase angle were greater than for the indicator of muscle functional quality. Significant logit models were obtained for the probability of occurrence of pre-sarcopenia, in which the strongest predictor was the phase angle, regardless of the type and number of covariates. The cut-off point of the phase angle for identification of pre-sarcopenia was 5.42° in men and 4.76° in women. Conclusion: The strong association between the risk of pre-sarcopenia and the phase angle, which can be easily and quickly assessed by bio-impedance analysis, suggests the necessity to include this parameter in routine geriatric evaluation in order to identify the risk of sarcopenia.

## 1. Introduction

The increasing aging of many populations, as well as the increase of unhealthy eating habits and global decline in physical activity, observed especially during the pandemic, will cause additional consequences forhealthcare systems already burdened with the incidence of COVID-19. An important challenge is the need to implement effective preventive and intervention programs, but also the evolution of geriatric assessment methods, including methods for identifying the risk of sarcopenia, towards improving and reducing the costs of diagnosis and treatment [1,2].

The 2018 European Working Group on Sarcopenia in Older People (EWGSOP2) revised its previous operational definition of sarcopenia (EWGSOP). Currently, sarcopenia is defined as a disease that causes skeletal muscle failure as a result of the loss of muscle strength and mass [3]. Previously, it was considered a geriatric syndrome, the main cause of which was the loss of skeletal muscle mass with age in the elderly, leading to a decline in muscle function [4]. Sarcopenia not only has serious health consequences such as weakness, disability, morbidity and mortality, but also high healthcare costs. The EWGSOP2 acknowledged in the revised consensus that, although sarcopenia is common among older adults, the development of sarcopenia begins in earlier periods, not only as a result of aging, but also as a result of chronic diseases, low protein intake and lack of physical activity. It is estimated that it increases with age from 3% in people under 65 to over 50% in people over 80 years of age. It is assumed that most cases of sarcopenia go undiagnosed [4,5,6].

The diagnosis is based on the evaluation of low muscle strength, which indicates the possibility of sarcopenia. The disease is confirmed iflow quantity or quality of muscles is found. In the case of diagnosed sarcopenia, its severe condition is identified by low physical fitness. EWGSOP2 recommended selected techniques for measuring individual parameters that constitute criteria for sarcopenia, but a gap was noticed in the availability of methods for assessing muscle quality. Muscle quality is expected to gain importance in defining sarcopenia [3], but this is not clearly defined because it covers many dimensions related to the structure, composition and functions of muscles, but is also relatedto the course of biochemical and involutional processes. Its most important indicators are believed to be the chemical composition and density of muscle tissue, as well as the type, number and distribution of muscle fibers, the presence of intramuscular adipose tissue, neuromuscular activation and aerobic capacity [7,8,9]. Currently, there is no standard protocol for assessing muscle quality. In scientific research, using sensitive imaging methods such as computed tomography and magnetic resonance imaging, the assessment of muscle quality is mainly focused on determining the density of the muscles and the presence of connective-fat tissue between the muscle bundles and fibers. High cost, the need for qualified staff and/or risk of harm to health with frequent exposure, limit the availability of these methods in routine clinical practice and screening [10].

The assessment of muscle condition can be assisted by Bioelectric Impedance Analysis (BIA), which is most often used to estimate body composition from prediction equations. Recording of raw impedance components, i.e., reactance, resistance and phase angle, gives us the opportunity to assess the electrical properties of tissues related to, among others, hydration and nutrition of cells, as well as structural and chemical changes [8,11,12,13]. Particular importance in assessing muscle quality is attributed to the bioelectrical impedance phase angle (PhA). This is a measure of the phase shift between voltage and current flowing through the tissues. A phase shift is a delay in the flow of current that is caused by the storage of the electrical charge in cell membranes. The value of the phase angle of impedance depends on the capacity of cell membranes, thus indirectly on the number and size of cells with integral membranes [11,14]. Higher PhA is a good predictor of a larger pool of intracellular water in the fluid distribution of the body and, consequently, a lower ratio of extracellular water to intracellular water (ECW/ICW) [12]. Reduction of the degree of cell hydration, which may lead to muscle atrophy, lowers the recorded values of the phase angle [11,13,14]. It is emphasized that the phase angle may have broad potential in assessing the nutritional status and stage of the disease, as well as in estimating the risk of postoperative complications, disability, and even mortality [14,15].

The aim of our study was to assess the risk of sarcopenia in people aged 50 and older, and its association with the bioelectrical impedance phase angle. We have made an attempt to determine whether the phase angle recorded as an electrical response of tissues in the BIA method could be a useful marker in identifying pre-sarcopenia in adults and the elderly.

## 2. Materials and Methods

### 2.1. Study Group and Project Details

The study included 1567 people (406 men and 1161 women) aged 50 to 87, who in 2010–2015 volunteered for free tests advertised in local media, health centers and associations of elderly people in south-west Poland. The inclusion criteria were aged 50 or more, as well as independence and autonomy in everyday life. Participants were evaluated to be subjectively healthy based on declarations of good health, no difficulty in walking, and no limitations in daily activities. None of the participants had a prior diagnosis of sarcopenia or its pre-clinical condition. Of the 1946 people registered for the tests, 379 were excluded. Exclusion criteria included limb amputation, the presence of metal prosthetic devices, electronically based implants, and a relative body mass index (BMI) greater than 50 kg/m^2^. Additionally, exclusion criteria included the usage of pharmaceutical based substances (hormones, corticosteroids) which could alter body composition. The study was approved by the Senate Research Ethics Committee of the Wroclaw University of Health and Sport Sciences (18 February 2009) and complied with the ethical requirements for human experimentation under the Helsinki Declaration. The participants were informed about the purpose and methods of the research, the procedures used and the experimental risks. All persons who declared participation in the study signed a voluntary and informed consent document. The project was financed by the Ministry of Science and Higher Education (grant no. N404 075337). The study was retrospectively registered on the ISRCTN registry (Ref: ISRCTN18225729; Date Registered 9 December 2020, https://doi.org/10.1186/ISRCTN18225729, accessed on 11 April 2022).

### 2.2. Anthropometric Measurements and Bioelectrical Impedance Analysis

Measurements of body weight and height were performed with an accuracy of 0.1 kg and 0.1 cm, respectively, on an electronic scale with a SECA 764 digital stadiometer (Seca GmbH & Co. KG., Hamburg, Germany). Body composition, including skeletal muscle mass, was estimated by bioelectric impedance analysis (BIA) using a TANITA MC 180 MA 8-electrode multi-frequency analyzer (Tanita Corporation, Tokio, Japan). The analyzer measures impedance with an accuracy of 0.01 Ω and phase angle with an accuracy of 0.01°. The values of resistance, reactance and phase angle were measured with an operating frequency of 50 kHz of amperage of 0.8 μA. The measurement was performed in standing position on a platform with four built-in electrodes (2 for each foot) and two two-electrode handgrips. Every day before the tests, the repeatability of the impedance measurements was checked in two consecutive trials in two volunteers. When registering for this study, participants were asked not to eat, drink or engage in any physical activity for at least three hours prior to the study, and to empty their bladder immediately before the measurement. Resistance (R), reactance (X_c_) and bioelectric impedance phase angle (PhA) were recorded for each participant.

Appendicular skeletal muscle mass (ASMM) was estimated, in accordance with the recent EWGSOP2 recommendations [3], using the prediction equation published by Sergi et al. [16]:

ASMM (kg) = −3.964 + (0.227 × Ht^2^/R) + (0.095 × Wt) + (1.384 × sex) + (0.064 × X_c_)

ASMM—appendicular skeletal muscle mass; Ht—height (cm); R—resistance (Ω); Ht^2^/R– resistance index (cm^2^/Ω); Wt—weight (kg); sex: men = 1 and women = 0; X_c_—reactance (Ω).

In order to minimize the differences related to inter-individual variability and because of the strong association between muscle mass and body size, the ASMM value was adjusted to the square of body height. Low muscle mass was identified for ASMM/Ht^2^ values <7.0 kg/m^2^ in men and <5.5 kg/m^2^ in women, sarcopenia cut-off points for criterion 2 [3].

### 2.3. Muscle Strength and Walking Speed Measurements

Hand grip strength (HGS) was measured with an accuracy of 1 kg using a JAMAR hydraulic dynamometer (Sammons Preston Rolyan, Bolingbrook, IL, USA) with an adjustable handle set to the 2nd position. The following recommendations of the American Society of Hand Therapists (ASHT) were applied: the subjectwas seated, the shoulder adducted and neutrally rotated, the elbow flexed at 90°, and the forearm and the wrist in a neutral position (the wrist between 0° and 30° of dorsiflexion) [17]. The subjects were asked to perform two maximum hand grip strength tests, alternating between the right and left hands, with each attempt lasting about 3–5 s, with a break between measurements of 15–20 s. During each measurement, the participant was motivated verbally to maximally squeeze the handle of the dynamometer: ‘Squeeze as hard as you can!’ The highest value of all attempts was recorded as the HGS value. Low muscle strength was identified for HGS < 27 kg in men and < 16 kg in women, sarcopenia cut-off point for criterion 1 [3]. The index of the measured hand grip strength values to the estimated appendicular skeletal muscle mass (HGS/ASMM) was taken as an indicator of the functional quality of muscles [3,7,9].

Walking speed (WS) was assessed using the 8-foot-up-and-go test from the Senior Fitness Test developed by Rikli and Jones [18]. The time taken to stand up from a sitting position, walk 8 feet to the marker, pivot, and return to a sitting position (total distance was 4.88 m) was measured. It was recommended to cover the distance as quickly as possible. The walking speed in meters per second was calculated for each participant. Low physical fitness was assumed as WS values < 0.8 m/s in men and women, the ‘adapted’ cut-off point for criterion 3 [3]. Due to the lack of validation of the 8-foot-up-and-go test used in this study for sarcopenia, its results WS < 0.8 m/s were considered only as an auxiliary criterion in assessing the severity of sarcopenia in individual cases, but did not affect the assessment of the prevalence of sarcopenia in the study population.

Participants were classified according to the EWGSOP2 algorithm for case-finding and assessment of the severity of sarcopenia [3]. Probable sarcopenia in the participants was found when criterion 1 (low muscle strength) was met, sarcopenia was confirmed when criterion 2 (low muscle quantity or quality) was additionally met, and severe sarcopenia was found if, in addition to the two previous criteria, criterion 3 was met (low physical performance). As the revised EWGSOP2 consensus [3] did not update the definition of early clinical and preclinical conditions, pre-sarcopenia was identified on the basis of low muscle mass (only criterion 2 met) in accordance with the previous EWGSOP consensus of 2010 [4]. Participants meeting only the second criterion of sarcopenia were qualified to the ‘pre-sarcopenia’ group, all the others to the ‘no sarcopenia’ group (criterion 2 not met). The pre-sarcopenia group was assessed as being at risk of sarcopenia.

### 2.4. Statistical Analysis

Basic descriptive statistics such as mean, standard deviation (SD) and confidence intervals (95% CI) were calculated for the results of all measurements. The percentage structure index was used to assess the occurrence of sarcopenia identified by the EWGSOP2 algorithm [3] and pre-sarcopenia identified by the EWGSOP consensus [4]. The normality of the distribution of all variables was checked by the Shapiro-Wilk test. Since the normal distribution was not confirmed for most of the variables, the non-parametric Kruskal-Wallis rank test and the χ^2^ test of independence were used to assess the differences between the sexes and the state of sarcopenia. Differences between non-Sarcopenia and pre-Sarcopenia in female and male groups were verified using the Mann-Whitney U test.

The probability of identifying pre-sarcopenia (pre-sarcopenia = 1; no sarcopenia = 0) was assessed by logistic regression. The independent variables were phase angle, gender (men = 1, women = 0), age, and body mass index. Reactance and resistance, which are trigonometric functions of the phase angle and were variables in the ASMM estimation equation, were not included in the analysis. Variables that were criteria for sarcopenia (HGS, ASMM, and WS) were also not included. Regression coefficients were tested using Wald Chi-square statistics. Variables that significantly correlated with the pre-sarcopenic state in univariate analyzes were included in multiple logistic regression (multivariate analysis). Using the stepwise technique, all selected variables were initially introduced into the model, and then those for which Wald’s *p* value exceeded 0.05 were eliminated. The number of variables was limited to non-interdependent variables to reduce the risk of redundancy. The goodness of fit of the logit model was checked with the Hosmer-Lemeshow (HL) test and assumed at *p* > 0.05. Based on the analysis of the receiver operating characteristic curie (ROC) and area under the curve (AUC), the diagnostic quality of PhA was assessed and the cut-off point for the pre-sarcopenia state was determined. All statistical analyzes were performed using TIBCO Statistica^®^ 13.3.0 (StatSoft Poland, Kraków, Poland). The statistical significance of the results was accepted at *p* < 0.05.

## 3. Results

**Figure 1:**
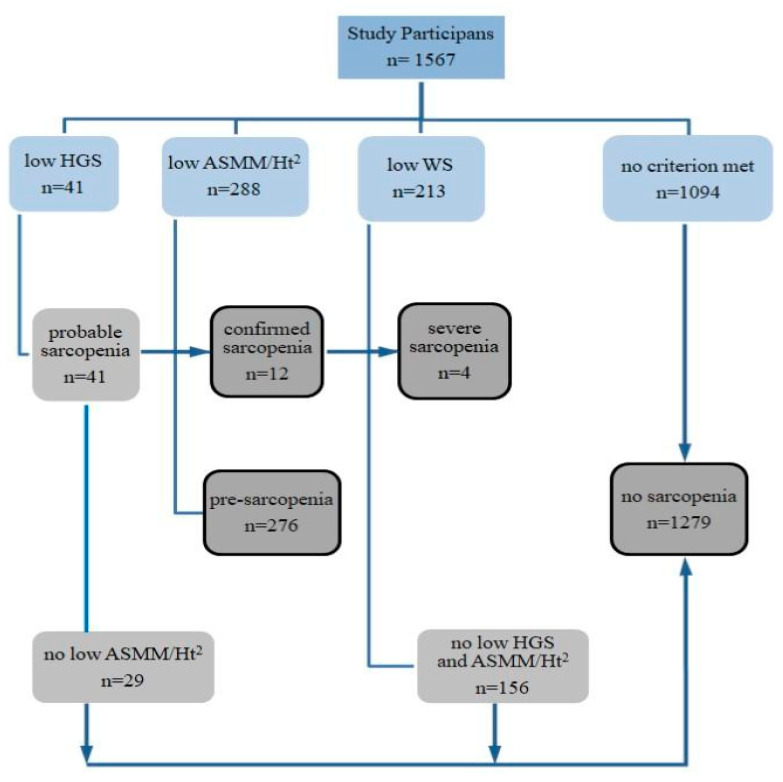
Scheme of identification and assessment of sarcopenia in participants of the study. HGS—hand grip strength, ASMM—appendicular skeletal muscle mass, Ht—height, WS—walking speed.

The results of the participants’ classification according to the criteria for sarcopenia identification are presented in Figure 1. Sarcopenia was diagnosed in 12 people (0.8%), including a likely severe condition in four (assessed on the basis of low walking speed). Among the diagnosed patients were 11 women, aged 60 to 83, and a 70-year-old man. Pre-sarcopenia, as assessed by low appendicular skeletal muscle mass adjusted to body height, was found in 18% of participants, 13% of whom were under 65 years of age.

Participants diagnosed with sarcopenia were excluded from further statistical analyzes due to insufficient numbers (*n* = 12). The results of measurements for this group were included in the Supplementary Materials (Table S1). The remaining participants were classified into two groups, one of which was people with no sarcopenia (*n* = 1279), and the other-people with a confirmed preclinical state of sarcopenia,‘pre-sarcopenia’ (*n* = 276). The descriptive characteristics of the study results, taking into account sex and the state of pre-sarcopenia, are presented in Table 1.

The Kruskal-Wallis test confirmed a significant differentiating effect of sex and sarcopenia state for most variables, except for body height and reactance, differentiated only by sex, and BMI differentiated by sarcopenia state. As expected, men compared with women were characterized by higher weight (Wt), body height (Ht), appendicular skeletal muscle mass (ASMM), hand grip strength (HGS), and they also had higher walking speed (WS), phase angle (PhA) and muscle functional quality index (HGS/ASMM). Reactance (X_c_) was higher in women than in men, and also in the case of X_c_ values adjusted to body height. The results of the tests for the significance of differences between men and women, which were expected and repeatedly reported in the literature, are not shown in the table.

**Figure 2:**
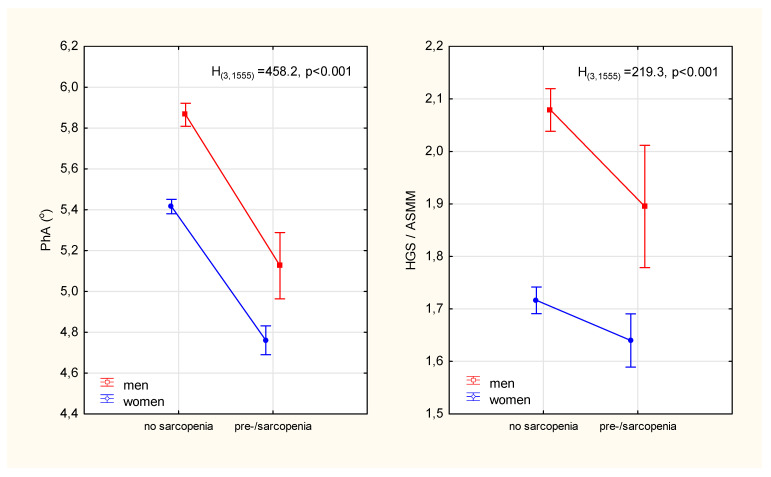
Differences in the phase angle of impedance and the index of the functional quality of the appendicular skeletal muscles between participants diagnosed with pre-sarcopenia and participants without sarcopenia. Points with vertical lines represent the mean with 95% confidence intervals; PhA—phase angle; HGS—hand grip strength; ASMM—appendicular skeletal muscle mass; H_(3,1555)_, p-Kruskal-Wallis rank ANOVA test results.

The prevalence of pre-sarcopenia in women was almost twice as high as in men (χ^2^ = 19.3, *p* < 0.001). In addition to the expected differences in age, weight, BMI and parameters that constituted the criteria for sarcopenia (i.e., muscle strength, mass and walking speed), it was observed that those identified as pre-sarcopenia compared to no sarcopenia had significantly lower phase angle values (Table 1). The relative percentage differences in PhA between the sarcopenic status groups were significantly higher (13% in men and 12% in women) than the differences in ASMM-adjusted strength (9% in men and 5% in women) (Figure 2)

The associations of selected variables (without their mutual interaction) with the probability of pre-sarcopenia state were initially checked using the method of univariate logistic regression. The variables that were the criteria for sarcopenia in this study were not taken into account. Age was a positive predictor of pre-sarcopenia, while sex, BMI, PhA, HGS/ASMM were negative predictors (Table 2). The phase angle most strongly determined the probability of sarcopenia risk. Increasing the PhA value by one unit reduces the chances of developing a pre-sarcopenia state by almost 17 times. For men, the chance of developing pre-sarcopenia was two times lower than the chance of developing it in women. Slightly lower reductions in odds were seen for a unit increase in BMI. Annual changes in age were the weakest determinant of the increase in the probability of pre-sarcopenia, but the logit results indicate that people 10 years older will be twice as likely to develop pre-sarcopenia than people younger than that (OR: 1.08^10^ = 2.15).

**Figure 3:**
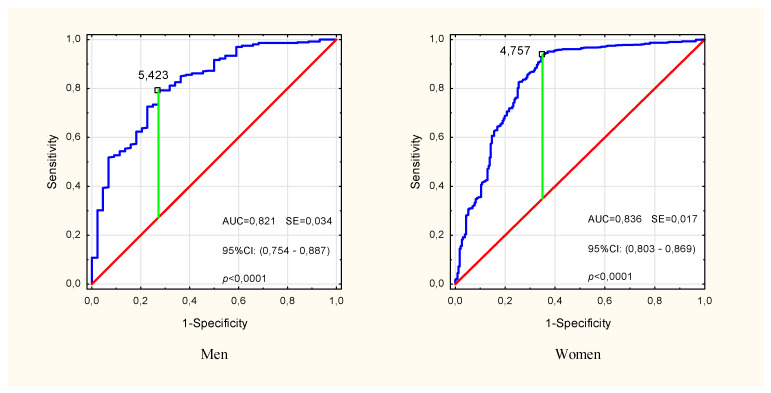
ROC curve for impedance phase angle in identifying pre-sarcopenia in males and females over the age of 50. The marked point is the cut-off point for the pre-Sarcopenia state. ROC—receiver operating characteristic, AUC—area under the ROC curve, SE—standard error, 95% CI—confidence interval.

In the multivariate modeling of the probability of pre-sarcopenia, the variables for which significance were confirmed in univariate analyzes were taken into account (Table 2). In the input model, sex (OR: 1.34, *p* = 0.18) and HGS/ASMM (OR: 0.82, *p* = 0.33) turned out to be insignificant factors in explaining the probability of pre-sarcopenia, and therefore were omitted in the subsequent stages of the analysis. Finally, age, BMI and PhA turned out to be the significant predictors (Multiple model 1 in Table 2). The sensitivity, specificity and accuracy of this model were 64.9%, 97.2% and 91.5%, respectively, but the differences between the expected and observed rates were significant (HL_(8)_ = 27.13, *p* < 0.001). Removal of BMI (Multiple model 2 in Table 2) improved the fit of the model (HL_(8)_ = 15.38, *p* = 0.052), lowering the sensitivity to 57.6%. Model 2 specificity and accuracy were 97.3% and 90.2%, respectively. In both models, the phase angle was the strongest predictor, and the presence of other covariates slightly changed its contribution to explaining the probability of pre-sarcopenia compared to the unadjusted (univariate) model. The analysis of the ROC curve showed a high classification performance of PhA (AUC = 0.821, *p* < 0.001 in men and AUC = 0.836, *p* < 0.001 in women) and the cut-off points for pre-sarcopenia PhA = 5.42° in men and PhA = 4.76° in women (Figure 3).

## 4. Discussion

In this study, we identified sarcopenia and its early clinical condition in almost 18% of participants declaring ‘good health’. In addition, we observed that pre-sarcopenia also occurred in participants under the age of 65. Cases of sarcopenia in adults after the age of 50 were recently reported from a UK Biobank cohort study of over 500,000 participants aged 37–73 [19]. The decline in muscle strength with age, which increases the risk of sarcopenia in adults, is determined to a greater extent by unfavorable changes in the condition of skeletal muscles than by the decline in muscle mass itself. The deterioration of muscle quality in the aging process is associated, among other factors, with a slowdown in metabolism, impaired neuromuscular activation and muscle contractile properties, the presence of connective-adipose tissue, changes in fiber distribution, and atrophy [2,9]. Structural and chemical changes occurring in muscle tissue affect its electrical properties, the registration of which using bioimpedance methods may facilitate the identification of changes in muscle quality [11,13].

In our study, the impedance phase angle differentiated the groups of no- and pre-sarcopenia more strongly than the indicator of functional muscle quality, which characterizes the strength generated by a unit of skeletal muscle mass. Participants with pre-sarcopenia had 12–13% lower PhA values compared to participants without sarcopenia. These differences, which took into account the age difference between groups, were significantly greater than the previously reported annual PhA losses of 1.6% in healthy elderly [20]. In a cross-sectional study of over 2000 adults aged 18–102, the estimated decline in PhA per decade of age was approximately 9% for men and 5% for women over 50 [21]. Estimated PhA values for healthy people between 50 and 80 years old gradually decrease from 6.5° to 5.6° for men and from 5.9° to 5.1° for women. Over the age of 80, the PhAs are 5.3° and 5.4° for men and women, respectively [15]. We observed significantly lower PhA than reference values in participants of this study with identified pre-sarcopenia: 5.13° and 4.76° in men and women aged ~70 years, respectively. The mean PhA values of participants without sarcopenia were within the limits estimated by Mattiello et al. based on a meta-analysis of 46 studies covering almost 250,000 people [15].

The decrease in the phase angle in the pre-sarcopenia group may be due to the smaller number and smaller size of cells with integral membranes which reduce the phase shift between the voltage and the current flowing through the tissues. The reduction in the size of cells is most often caused by a decrease in their hydration and nutrition, and a reduction in the lipid content of cell membranes, which in turn leads to atrophy of muscle cells. Due to its sensitivity to changes in cell mass and the distribution of intra- and extracellular fluids, the phase angle is considered a qualitative measure of soft tissues [14]. Significant reductions in the phase angle correlating with loss of skeletal muscle mass, strength and functional quality in the elderly have been reported in many previous studies [13,20,22].

The contribution of the phase angle in explaining the variability of parameters important in the diagnosis of sarcopenia suggests that it could be a good marker in the assessment of the risk of its occurrence. It has been found that low phase angle values are a good predictor of limitations in daily activities and may increase the risk of disability later in life [23,24]. Higher PhA values were associated with better physical fitness, greater muscle strength, agility, and dynamic balance control in healthy elderly people, regardless of sex, age, and skeletal muscle mass [25,26]. The phase angle was also used in the prognosis of functional outcomes after postoperative rehabilitation in patients after hip fracture [27]. Slee et al. [22] showedan association between low phase angle and malnutrition and frailty syndrome. In a recent study by Kołodziej et al. [28], it was confirmed that a lower phase angle in the elderly correlates with an increased risk of physical frailty, the development of which is most often caused by sarcopenia [3].

In a systematic review that took into account studies published between 2012 and 2020 with a total of 7668 participants, Di Vincenzo et al. [29] reported that the phase angle was significantly reduced in sarcopenia compared to non-sarcopenia, in both general populations and in patients with various clinical conditions. The results of the review suggest that a reduction in PhA was also identified in people with pre-sarcopenia, although there is little research into its association with PhA. It was confirmed that the prevalence of sarcopenia was higher in people with low PhA, but reported PhA cut-off values differed between studies due to differences between populations, the criteria used to identify sarcopenia, and methods for determining diagnostic thresholds [29]. The good predictive ability of PhA for sarcopenia was confirmed in a recent study of young and old people in Japan. For the elderly, the cut-off points were PhA = 5.04° in men (age 74.4 ± 5.5 years) and PhA = 4.20° in women (age 73.1 ± 6.4 years) [30]. Although a decrease in phase angle was reported in cases of pre-sarcopenia [29], to our knowledge there is no data regarding PhA limit values for identifying this condition in general populations.

In our study of subjectively healthy people aged 50 and above, we found a significant association of low phase angle with the likelihood of developing pre-sarcopenia, which did not lower significantly even with confounding variables such as sex, age and BMI. Moreover, the involvement of PhA (sex dependent) in predicting the state of pre-sarcopenia significantly reduced the role of sex, whose predictive ability for sarcopenia has not yet been fully assessed. Epidemiological data regarding the sex differences in the prevalence of sarcopenia are conflicting. Patel et al. [6] in a study of 1890 elderly people (868 men and 1022 women), reported the prevalence of sarcopenia at the level of 4.6% in men and 7.9% in women. Bijlsma et al. [5] in a study of 325 men and 329 women, reported the prevalence of sarcopenia of 4.6% and 2.1%, respectively. In women, an additional factor that increases the risk of sarcopenia is younger age at menopause. This has been suggested to be related to muscle redistribution and fat accumulation in the form of intramuscular fat in menopausal women [31]. Sex disproportions in individual age groups could have influenced our observed by frequency of pre-sarcopenia as twice as high in women than in men. In both sex groups, low PhA values (≤5.42° for men and ≤4.76° for women) had good accuracy in predicting pre-sarcopenia (AUC > 0.82).

Given the trend of PhA to decrease with age by approximately 0.8–0.9° between the ages of 50 and 80 [15], our PhA cut-off points for pre-sarcopenia may have limited applicability, depending on the age of the target population. Additionally, they can only be used for PhA values measured at 50 kHz. The sensitivity of measuring instruments, the arrangement of electrodes and the position of the examined person may also be important [15]. Another limitation of our study was the lack of assessment of the association of the phase angle with sarcopenia defined in accordance with the EWGSOP2 revised consensus. The reason for this was the insufficient number of cases with confirmed sarcopenia in the population of people declaring good health. Although our results can only be applied to the state of pre-sarcopenia, they confirm the need to monitor the biological condition in terms of the prevention of sarcopenia, not only in the elderly, but also in earlier periods of life. The use of ASMM values estimated by BIA for sarcopenia criterion 2 may also be debatable, but we wanted to point out the potential of this method in routine monitoring of the aging process as an alternative to limited available reference methods such as computed tomography, magnetic resonance or dual energy x-ray absorptiometry. In addition, the method itself and the ASMM prediction equation we used, developed by Sergi et al. [16], is taken into account in the latest EWGSOP2 recommendations [3].

The advantage of our research is the large number of people who live independently, who are subjectively healthy and ethnically homogeneous. The results obtained from studies in this population further support the EWGSOP2 call for early prevention of sarcopenia and focus in research on measures of muscle quality as an important parameter in defining sarcopenia [3]. We believe that the risk of sarcopenia can be assessed using the simple, fast and non-invasive BIA method, which will enable early application of therapeutic methods to prevent the development of sarcopenia in adults and the elderly.

## 5. Conclusions

The presented research results confirm that a low phase angle predicts the risk of pre-sarcopenia. We believe that PhA, recognized as a measure of muscle cellular quality, may be a helpful marker in assessing the risk of sarcopenia, its severity, and treatment progress in adults and the elderly in routine clinical practice, as well as in preventive and screening tests. The use of the prognostic potential of the phase angle, recorded with the available and cheap BIA method, would improve the identification of adverse changes in the condition and function of skeletal muscles in order to prevent sarcopenia. Such a possibility creates the need to determine the reference and normalized cut-off points of the phase angle value for sarcopenia.

## Figures and Tables

**Table 1:** Descriptive characteristics of study participants.

Men				Women		
	No Sarcopenia (*n* = 361)	Pre-Sarcopenia (*n* = 44)		No Sarcopenia (*n* = 918)	Pre-Sarcopenia (*n* = 232)	
	Mean ± SD (95% CI)	Mean ± SD (95% CI)	*p*	Mean ± SD (95% CI)	Mean ± SD (95% CI)	*p*
Age (years)	65.7 ± 6.6 (65.0–66.4)	72.1 ± 6.9 (70.0–74.2)	<0.001	64.2 ± 5.9 (63.8–64.6)	67.3 ± 7.8 (66.3–68.3)	<0.001
Ht (cm)	173.7 ± 6.3 (173.0–174.3)	172.6 ± 5.4 (170.9–174.2)	0.330	159.3 ± 5.9 (158.9–159.6)	158.9 ± 5.9 (158.2–159.7)	0.526
Wt (kg)	87.6 ± 12.2 (86.3–88.9)	75.9 ± 8.4 (73.3–78.4)	<0.001	73.4 ± 11.5 (72.7–74.2)	60.5 ± 9.0 (59.3–61.7)	<0.001
BMI (kg/m^2^)	29.0 ± 3.6 (28.6–29.4)	25.5 ± 2.6 (24.7–26.3)	<0.001	28.0 ± 4.3 (28.7–29.2)	24.0 ± 3.5 (23.5–24.4)	<0.001
HGS (kg)	47.6 ± 9.7 (46.6–48.6)	38.1 ± 9.0 (35.3–40.8)	<0.001	27.8 ± 6.2 (27.4–28.2)	22.3 ± 6.2 (21.5–23.1)	<0.001
WS (m/s)	1.02 ± 0.16 (1.01–1.04)	0.97 ± 0.17 (0.92–1.02)	0.022	0.90 ± 0.16 (0.89–0.91)	0.87 ± 0.15 (0.85–0.89)	0.011
PhA (°)	5.87 ± 0.59 (5.80–5.93)	5.13 ± 0.57 (4.95–5.30)	<0.001	5.42 ± 0.55 (5.38–5.45)	4.76 ± 0.48 (4.70–4.82)	<0.001
X_c_ (Ω)	50.9 ± 7.6 (50.1–51.7)	49.1 ± 6.7 (47.1–51.2)	0.196	57.9 ± 8.4 (57.4–58.5)	59.2 ± 7.7 (58.2–60.2)	0.039
ASMM (kg)	23.0 ± 2.3 (22.7–23.2)	20.2 ± 1.8 (19.7–20.8)	<0.001	16.3 ± 1.9 (16.1–16.4)	13.7 ± 1.4 (13.5–13.9)	<0.001
ASMM/Ht^2^ (kg/m^2^)	7.61 ± 0.58 (7.55–7.67)	6.78 ± 0.41 (6.66–6.91)	<0.001	6.41 ± 0.64 (6.37–6.45)	5.43 ± 0.44 (5.37–5.48)	<0.001
HGS/ASMM	2.08 ± 0.40 (2.04–2.12)	1.90 ± 0.46 (1.76–2.03)	0.023	1.72 ± 0.37 (1.69–1.74)	1.64 ± 0.46 (1.58–1.70)	0.041

SD—standard deviation, 95% CI—confidence interval, Ht—height, Wt—weight, BMI—body mass index, HGS—hand grip strength, WS—walking speed, PhA—phase angle, X_c_—reactance, ASMM—appendicular skeletal muscle mass, HGS/ASMM—muscle quality index.

**Table 2:** Logistic regression models for the likelihood of pre-sarcopenia in adults after 50 years of age.

	Predictor	Coefficient Estimate	SE	OR (95%CI)	Wald Statistic	*p*-Value
Univariate models	Sex	−0.73	0.18	0.48 (0.34–0.68)	17.20	<0.001
	Age	0.08	0.01	1.08 (1.06–1.10)	58.80	<0.001
	BMI	−0.44	0.03	0.65 (0.61–0.68)	217.0	<0.001
	PhA	−2.89	0.19	0.06 (0.04–0.08)	240.9	<0.001
	HGS/ASMM	−0.81	0.17	0.45 (0.32–0.62)	23.48	<0.001
Multiple model 1	Intercept	19.48	1.78		119.6	<0.001
	Age	0.07	0.01	1.07 (1.04–1.10)	23.51	<0.001
	BMI	−0.48	0.04	0.62 (0.58–0.67)	161.9	<0.001
	PhA	−2.55	0.21	0.08 (0.05–0.12)	143.2	<0.001
Multiple model 2	Intercept	11.0	1.37		64.64	<0.001
	Age	0.03	0.01	1.03 (1.00–1.05)	5.08	<0.024
	PhA	−2.78	0.19	0.06 (0.04–0.09)	212.7	<0.001

Sex: men = 1, women = 0; BMI—body mass index, PhA—phase angle; SE—standard error; OR—odds ratio; 95% CI—confidence interval.

## Data Availability

The data presented in this study are available on request from the corresponding author. The data are not publicly available due to ethical restrictions.

## Supplementary Materials

The following supporting information can be downloaded at: https://www.mdpi.com/article/10.3390/ijerph19084687/s1, Table S1. Measurement results of participants with identified sarcopenia (1 man and 11 women).
