# Supplementary material for: The Use of the Bioelectrical Impedance Phase Angle to Assess the Risk of Sarcopenia in People Aged 50 and above in Poland
Source: Int J Environ Res Public Health. 2022 Apr 13;19(8):4687. doi: 10.3390/ijerph19084687 (PMC9031980; doi:10.3390/ijerph19084687)
Supplement: Supplementary file 1 [file ijerph-19-04687-s001.zip › ijerph-1670018-supplementary.pdf]

Supplementary material to: The use of the bioelectrical impedance phase angle to assess the risk of sarcopenia in people aged 50 and above in Poland

Table S1. Measurement results of participants with identified sarcopenia (1 man and 11 women).

	**Mean ± SD**
Age (years)	72.7 ± 8.3
Ht (cm)	155.9 ± 5
Wt (kg)	58.8 ± 6.7
BMI (kg/m^2)	24.2 ± 2.4
HGS (kg)	14.3 ± 2.6
WS (m/s)	6.55 ± 1.22
PhA (°)	4.64 ± 0.38
Xc(Ω)	55.1 ± 5.8
ASMM (kg)	13.5 ± 1.8
ASMM/Ht^2 (kg/m^2)	5.52 ± 0.47
HGS/ASMM	1.07 ± 0.12

SD – standard deviation, Ht– height, Wt– weight, BMI – body mass index, HGS – hand grip strength, WS – walking speed, PhA – phase angle, Xc – reactance, ASMM – appendicular skeletal muscle mass, HGS/ASMM – muscle quality index.
